# Dilemma of Treating Psychosis Secondary to Stroke

**DOI:** 10.7759/cureus.12763

**Published:** 2021-01-18

**Authors:** Ruchita Agrawal, Shikha Verma, Vatsalya Vatsalya, Monica Halappanavar, Kosisochukwu Oraka

**Affiliations:** 1 Psychiatry, Seven Counties Services, Louisville, USA; 2 Psychiatry and Behavioral Health, Rosalind Franklin University, North Chicago, USA; 3 Child and Adolescent Psychiatry, Rogers Behavioral Health, Kenosha, USA; 4 Medicine, University of Louisville School of Medicine, Louisville, USA; 5 Psychiatry and Behavioral Sciences, University of Louisville, Louisville, USA; 6 Medicine, Vinnytsia National Medical University, Vinnytsia, UKR

**Keywords:** stroke, post stroke psychosis, long term neuropsychiatric sequelae, psychosis

## Abstract

Post-stroke psychosis is prevalent and disabling with increased mortality risk. Treatment for post-stroke psychosis is limited in this staggering medical concern. The most commonly used medications are antipsychotics, however, the risk for stroke increases further with the use of antipsychotics. Furthermore, interventional clinical studies have not been carried out to test the efficacy and safety of antipsychotics in the management of post-stroke psychosis. We present a case of post-stroke psychosis to highlight the risks faced by these patients in terms of daily function and safety concerns and the challenges encountered in treatment due to poor response to the conventional antipsychotics; and so calling attention to early diagnosis and improved treatment options. More clinical investigations are needed to address the pathology associated with the clinical presentation and exploring the pharmacotherapies to improve efficacy and safety of treatment for post-stroke psychosis.

## Introduction

Stroke is the second most common cause of mortality and the fourth leading cause of disability worldwide [1,2]. Neuropsychiatric symptoms following stroke occur in at least 30% of stroke survivors. As per a recent systematic review, about 4.86% of the patients have delusions or hallucinations secondary to stroke. Most commonly, lesions are in the frontal, temporal, and parietal regions [3].

Patients with post-stroke psychosis are 51% more likely to die in ten years, as compared to patients with no psychosis after stroke [4]. Among delusions, persecutory delusions are found to be more common in post-stroke psychosis; and delusional denial of illness (anosognosia) is found most commonly in patients with the right hemisphere stroke [5,6]. Studies have shown an increased risk of psychosis with a family history of psychosis, traumatic brain injury (TBI) in adolescence, in severe TBI and temporal lobe lesions [7,8].

Psychosis occurs due to the dysfunction of subcortical basal ganglia limbic system interaction, involving abnormal dopaminergic neurotransmitters. The content of delusions can reflect the underlying function of the cortex. Psychosis can develop within the first few days following a stroke or can have delayed-onset by several weeks or months [9]. The risk of developing psychosis, after moderate to severe TBI, is at most two years after injury [4]. For some patients, psychosis is transient and resolves on its own, however, it does not in others (without any indication of resolution with long-term care). Psychosis is seen in about 3.3% of stroke inpatients [10]. Antipsychotics are the most common treatment modality for post-stroke psychosis [11]. When compared with the general population, studies have shown that there could be a 50% increased risk of stroke with antipsychotics by first-generation antipsychotics (FGA), and this risk decreases to 30% with second-generation antipsychotics (SGA) [9].

We present a case report of post-stroke psychosis in a patient with basal ganglia lesion whose psychosis did not improve with conventional antipsychotic treatment. The question remains, what is the best treatment for patients with post-stroke psychosis who do not respond to conventional antipsychotics.

## Case presentation

A 48-year-old Caucasian male with no prior personal or family history of mental health diagnosis presented with new-onset psychosis, status post-ischemic stroke after motor vehicle accident (MVA). He suffered from an MVA in which he was an unrestrained passenger sustaining a head-on collision without loss of consciousness. He was heavily intoxicated by alcohol. Workup during hospitalization identified a fracture of the right femur in radiological reports. He had open treatment of femoral supracondylar fracture with intercondylar extension. The patient’s urine screened positive for tetrahydrocannabinol (THC), and opioids prescribed were prescribed to the patient.

The patient suffered from a stroke one week after the MVA. The stroke resulted in right-sided limb weakness, dysarthria, and right facial droop. Psychosis started a few days after the stroke. Symptoms included auditory hallucinations (AH), persecutory delusions about being watched through cameras, thought broadcasting through microphones, having ideas of reference about receiving special messages through television. In 2017, he set his house on fire and injured his father secondary to commanding AH, leading to legal charges against him. He frequently felt aggravated as a result of the commanding AH and at times, also acted on those commands. He described feeling depressed when not angry or irritable. He has not been employed since the MVA. Sleep, memory, and ability to concentrate are poor due to ongoing AH. His medical history includes stroke in 2016, asthma, heart implant, intramedullary rods in both legs, limping gait, arthritic changes in the right knee, and right elbow injury. History of substance use was clinically significant for methamphetamine abuse that started two months after the MVA. He remained sober from alcohol for over a year at the time of the report, however used tobacco dips regularly. Family history was significant for stroke in his paternal lineage (father and grandfather); and no known family history of mental health diagnosis.

The patient was initiated on the trial of olanzapine and divalproex and doses were titrated slowly to olanzapine 20mg every night (qHS) and divalproex 500mg twice daily (BID). These medications did not resolve the symptoms of psychosis substantially. The positive and negative syndrome scale (PANSS) score before initiation medicines was 180, and after adequate dose trial decreased to 147. Therefore, combinatorial pharmacogenomics testing was performed to guide the treatment of choice for psychotropic medication regimen. Test results did not indicate any gene interaction with the majority of antipsychotics. Among selective serotonin reuptake inhibitor (SSRI), fluoxetine and paroxetine were predicted to have moderate and severe interactions, respectively. However, other antidepressants showed no significant interactions. The human serotonin transporter gene (SLC6A4) showed a normal response, and the patient was noted to be homozygous for long promoter polymorphism of serotonin transporter. Serotonin receptor 2A (HTR2A) homozygous variant for allele concluded an increased risk of adverse reaction with SSRI. Human leukocyte antigen (HLA)-b*1502 and HLA-a*3101 has low risk, as they do not carry this allele.

## Discussion

Our patient sustained basal ganglia lesion due to stroke and developed psychosis a few days later. As documented in previous studies, post-stroke psychosis secondary to lesions in basal ganglia is common. Primary complaints were mood changes, persecutory delusions, and commanding auditory hallucinations. As compared to patients with schizophrenia, he had good insight into his symptoms and there were no negative symptoms. Basal ganglia is a dopamine rich region and so becomes the target of antipsychotic medicines [12]. Despite being maintained on a therapeutic dose of antipsychotic, our patient's psychosis did not improve completely. The patient’s genetic testing does not reveal any significant gene interaction or poor metabolism with psychotropic medications. Since the risk of stroke increases with antipsychotics, the course of this patient’s etiological intervention and medical management should be of primary concern. The immediate treatment module is to either add another antipsychotic; or to switch to some other second-generation antipsychotic or first-generation antipsychotic. No medication is food and drug administration (FDA) approved for post-stroke psychosis. One case report discussed improvement in symptoms with the combination of clozapine and divalproex, however, another study remains mechanistically (therapeutic-pathway target) inconclusive [13,14]. More clinical studies and longitudinal therapeutic investigations are needed to address the gaps discussed in this case report.

## Conclusions

Post-stroke psychosis can be complicated to treat due to available treatment options and risks involved with medications. It can have a huge impact on the quality of life and increased the disease burden. The mortality rate for patients with post-stroke psychosis is much higher. Thus, it is also important to identify at-risk patients who may develop psychosis after a stroke. Limited benefits of existing treatment options also indicate that process of psychosis in post-stroke individuals might be different than psychosis in individuals with no stroke. More clinical investigations are needed to address the pathology associated with the clinical presentation, to develop specific tools that can assist in expanding on preventive measures, and to explore the pharmacotherapies to improve efficacy and safety of treatment for post-stroke psychosis.
